# How Soon after Exposure to Sepsis May the Accoucheur Resume Practice?

**Published:** 1885-08

**Authors:** 


### How Soon after Exposure to Sepsis may the Accoucheur Resume Practice?

The great bulk of obstetricians and surgeons to-day, among whom may be mentioned Emmett, Battey, Goodell and Thomas in this country, and Martin, Schroeder, Volkmann, Nussbaum and Esmarch in Europe, believe that time is not essential, and that thorough disinfection is only necessary. So confident is Volkmann of the truth of his belief, that from work upon the cadaver he goes to operations of all sorts, and from fetid abscesses and erysipelatous cases he goes directly to obstetric cases. He says, unconditionally, "I have never infected a patient."

